# Supplementary material for: Non-Hermitian route to higher-order topology in an acoustic crystal
Source: Nat Commun. 2021 Mar 25;12:1888. doi: 10.1038/s41467-021-22223-y (PMC7994416; doi:10.1038/s41467-021-22223-y)
Supplement: Supplementary file 1 — Supplementary Information [file 41467_2021_22223_MOESM1_ESM.pdf]

Supplementary Information for
Non-Hermitian route to higher-order topology in an acoustic crystal

He Gao[1], Haoran Xue[2⋆], Zhongming Gu[1], Tuo Liu[1], Jie Zhu[1,3⋆] and Baile Zhang[2,4⋆]

[1]Department of Mechanical Engineering, The Hong Kong Polytechnic University, Hung Hom, Kowloon, Hong Kong SAR, China.
[2]Division of Physics and Applied Physics, School of Physical and Mathematical Sciences, Nanyang Technological University, Singapore 637371, Singapore.
[3]The Hong Kong Polytechnic University Shenzhen Research Institute, Shenzhen 518057, China.
[4]Centre for Disruptive Photonic Technologies, Nanyang Technological University, Singapore 637371, Singapore.
⋆e-mail: haoran001@e.ntu.edu.sg; jie.zhu@polyu.edu.hk; blzhang@ntu.edu.sg.

Supplementary Note 1. Extracted loss parameters in a single resonator

Here we present measurements on a single resonator without or with additional losses. The resonator has a dipole mode at 2139.6 Hz, whose mode profile is shown in Supplementary Figure 1a. For a single resonator without additional losses, the measured spectrum has a peak at around 2139 Hz, as denoted by the red dot in Supplementary Figure 1b. By fitting the experimental spectrum to the blue line, we can take the background loss into consideration in simulation by setting sound speed to be $342.34 + 2.23i$ m/s. We have also measured the spectrum for the resonator with additional losses, as shown in Supplementary Figure 1c. The spectrum becomes broader while the peak frequency remains almost unshifted. Similarly, the sound speed is estimated to be $342.34 + 14.72i$ m/s. These values are then used in numerical simulations for the bulk dispersion and eigenstates of finite lattices.

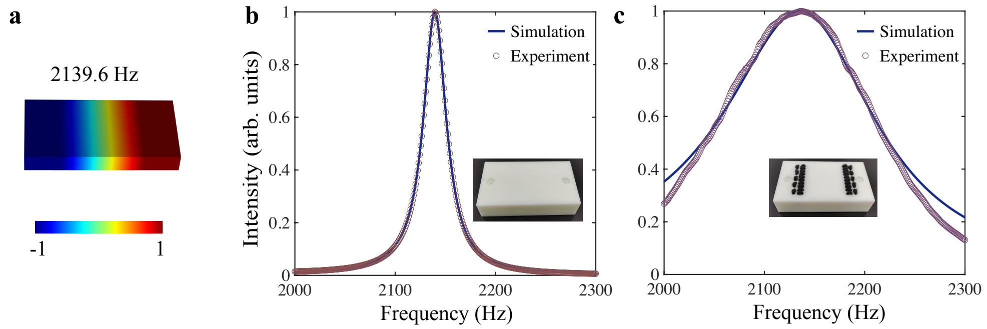

Supplementary Figure 1 Measured spectra for the single resonators. **a**, Eigenmode for the dipole mode of our interest in this work. **b**, Measured spectrum for a single resonator without additional losses. **c**, Measured spectrum for a single resonator with additional losses. The red dots denote measured results and the blue curves are the numerically fitted results. The insets are the photos of the printed single resonators.

Supplementary Note 2. Tight-binding model and Wannier bands

The physics of our coupled acoustic resonator system can be well-captured by a tight-binding model which only includes the mode of our interest and nearest-neighbor couplings.[1] The tight-binding Hamiltonian corresponding to the lattice shown in Fig. 1c in the main text is

$$\begin{aligned} H(\mathbf{k}) =& \kappa\sigma_0\sigma_z\sigma_0\sigma_x - \kappa\sigma_0\sigma_x\sigma_0\sigma_0 + [(1-i\gamma_1)\sigma_0\sigma_0\sigma_0\sigma_0 + i\frac{\gamma_2-\gamma_1}{2}(\sigma_z\sigma_z\sigma_z\sigma_z - \sigma_0\sigma_0\sigma_0\sigma_0)]f_0 \\ &+ \kappa\sigma_0[\sigma_z(\sigma^-\sigma^+ + e^{-ik_xa}\sigma^-\sigma^- + \text{H.c.})] - \kappa(\sigma^-\sigma^+\sigma_0\sigma_0 + e^{-ik_ya}\sigma^-\sigma^-\sigma_0\sigma_0 + \text{H.c.}). \end{aligned} \quad (1)$$

Here κ is the coupling amplitude and a is the lattice constant, $\sigma_{x,y,z}$ are the Pauli matrices, σ_0 is the identity matrix and $\sigma^{+,-} = (\sigma_x \pm i\sigma_y)/2$. f_0 is the resonant frequency of a single resonator. γ_1 and γ_2 correspond to the background loss and

the enhanced loss, respectively. H.c. stands for Hermitian conjugate. By fitting the bandstructure given by Eq. S1 to the one obtained from full-wave simulation, we obtain the tight-binding parameters as: κ =17.08 Hz, f_0=2139.6 Hz, γ_1=0.0065 and γ_2=0.0415. As can be seen in Figs. S2c and d, the bandstructure given by full-wave simulation (Supplementary Figure 2c) agrees well with the one calculated from the tight-binding model (Supplementary Figure 2d). In contrast, for the lattice without the additional loss ($\gamma_2 = \gamma_1$), the full-wave (Supplementary Figure 2a) and tight-binding (Supplementary Figure 2b) bulk bands are gapless. This demonstrates that the additional loss opens up a bandgap. In our design, the induced bandgap is around 45 Hz. A larger bandgap can be achieved by increasing coupling strength κ and additional loss γ_2. Moreover, extending the design to phononic crystal structures that go beyond tight-binding model may also yield a larger bandgap.

In the main text, we have shown the eigenfrequencies of a finite lattice when γ_2=0.0415. Here we plot in Supplementary Figure 2e the evolution of the eigenfrequencies as a function of $\gamma_2 - \gamma_1$ based on the tight-binding model. As can be seen, a bandgap is opened and in-gap corner states emerge as $\gamma_2 - \gamma_1$ is increased from zero. The red dashed line denotes the parameter value we used in experiment. At this value (γ_2=0.0415), the bandgap is relatively large and the edge states are slightly away from the bulk states, which are all good for the measurements.

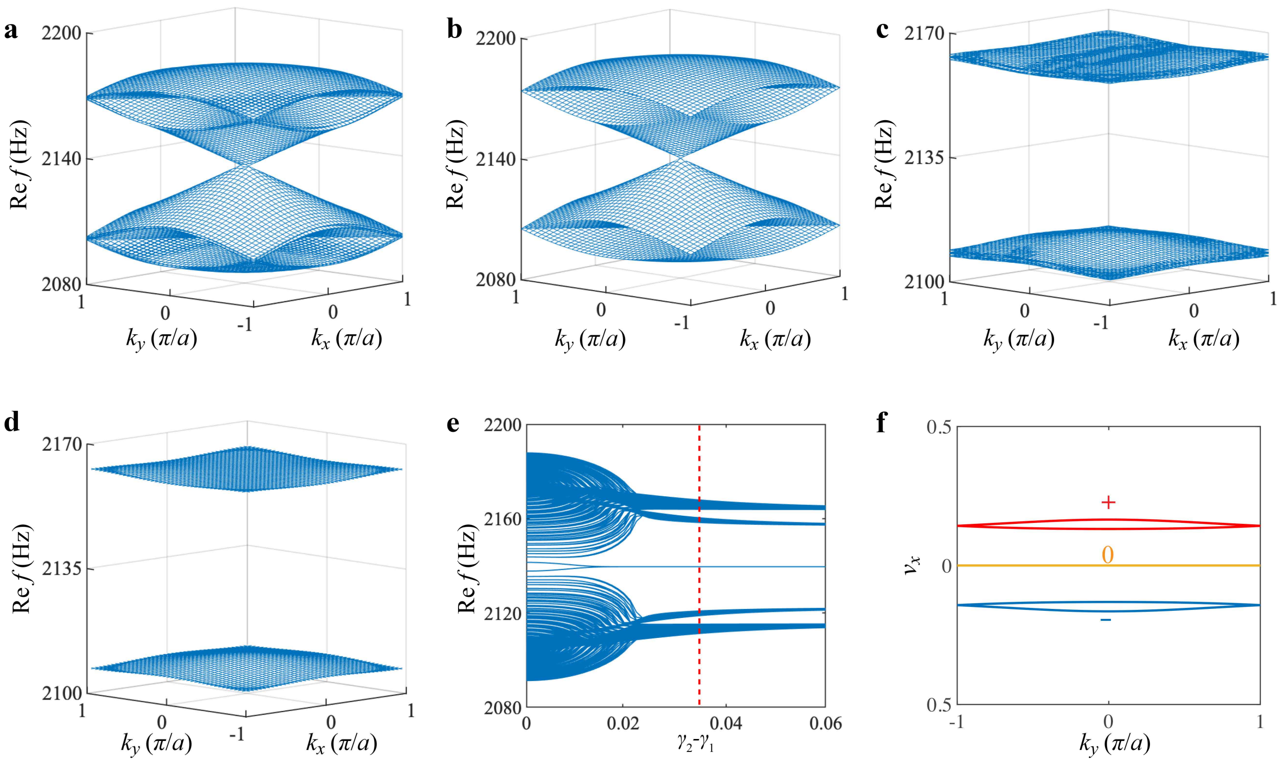

Supplementary Figure 2 Bandstructures and Wannier bands for the nontrivial lattice. **a**, Bulk bandstructure for the acoustic lattice with a uniform background loss calculated from full-wave simulation. **b**, Bulk bandstructure for the acoustic lattice with a uniform background loss calculated from tight-binding model. **c**, Bulk bandstructure for the nontrivial lattice illustrated in Fig. 1c in the main text calculated from full-wave simulation. **d**, Bulk bandstructure for the nontrivial lattice illustrated in Fig. 1c in the main text calculated from tight-binding model. **e**, Calculated eigenfrequencies for a 40×40 finite lattice as a function of the $\gamma_2 - \gamma_1$. The red dashed line indicates the parameter value used in experiment. **f**, Wannier bands ν_x calculated from the tight-binding model for $\gamma_2 = 0.0415$.

Next we calculate the Wannier bands and Wannier sector polarization with the tight-binding model. In non-Hermitian systems, the eigenstates are in general nonorthognal. However, they can be biorthogonalized as $\langle u_m^L(\mathbf{k})|u_n^R(\mathbf{k})\rangle = \delta_{mn}$ (m, n represent the occupied bands and L/R denote the left/right eigenvectors). Under the biorthogonal condition, we can use the nested Wilson loop approach to characterize the nontrivial topology.[2] First, we define a Wilson loop operator along x direction as:

$$W_x(k) = F_x(k + N_x\Delta k_x)\cdots F_x(k + \Delta k_x)F_x(k). \tag{2}$$

Here $F_x(k)$ is a 8×8 matrix with elements defined by $[F_x(k)]^{mn} = \langle u_m^L(k + \Delta k_x)|u_n^R(k)\rangle$. N_x is the number of sampling points and $\Delta k_x = 2\pi/a/N_x$. The Wannier Hamiltonian is given by $H_{W_x}(k) = -\frac{i}{2\pi}\log(W_x(k))$. The eigenvalue spectrum of $H_{W_x}(k)$ is called Wannier bands. The calculated Wannier bands for our system are plotted in Supplementary Figure 2f, which consist of three separated sectors denoted by "+", "-" and "0". The spectrum is symmetric with respect to zero as required by mirror symmetries, yielding a vanishing bulk polarization. To see the quadrupole topology, we further calculate

a nest Wilson loop over a certain Wannier sector by defining the biorthogonal subspace:

$$|w_x^{\alpha,L/R}(k)\rangle = \sum_{n=1}^{8} |u_n^{L/R}(k)\rangle [v_x^{\alpha,L/R}(k)]^n, \tag{3}$$

where $v_x^{\alpha,L/R}(k)$ are the eigenstates of the Wannier Hamiltonian and α refers to a certain Wannier sector. The Wannier sectors "+" and "-" carry a nontrivial polarization of 0.5, which indicates the edge is a topological insulator with quantized dipole moment that is induced by a quantized bulk quadrupole moment.

Supplementary Note 3. Position choice of positive/negative coupling

To realize couplings with opposite signs, we design two different coupling configurations[1] as shown in Supplementary Figure 4a and b, respectively. The coupling waveguides connect to different sides of the resonance's nodal line in these two configurations, thus realizing couplings with opposite signs. This can be directly seen through the eigenmode profiles. In Supplementary Figure 4a and b, the two eigenmodes switch their eigenfrequencies as we change one configuration to the other, indicating the flip of coupling sign.

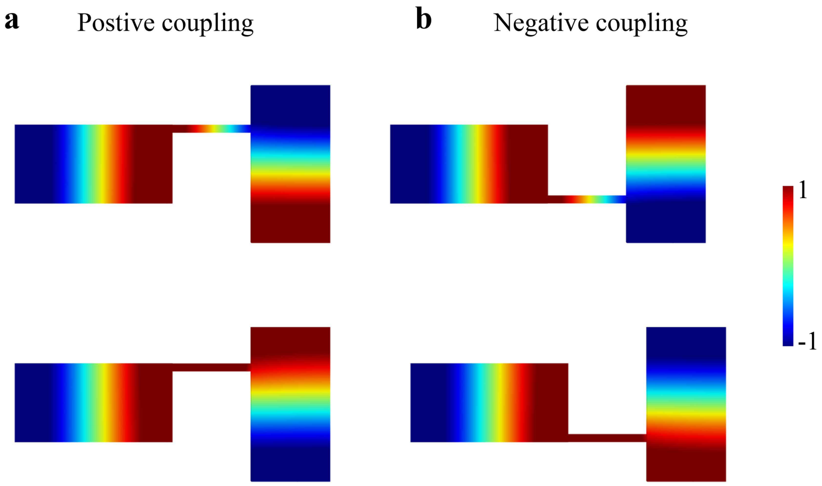

Supplementary Figure 3 Eigenmodes for two coupled acoustic resonators. Eigenmodes of two coupled resonators with positive coupling (**a**) and negative coupling (**b**), respectively.

Supplementary Note 4. Details for the experimental setup

In each resonator, two small air holes are drilled at two sides that allow for the signal input and output in experimental measurements. These small holes can be openned or closed by the two circular covers. During the measurements, the input source and microphone are at the two air holes to excite and detect the response of the same resonator. The lock-in amplifier (Zurich Instrument HF2LI) is connected to the computer, which acts as signal generator and data acquisition system simultaneously. The incident sound waves were generated by a loudspeaker with a swept signal ranging from 2000 Hz to 2300 Hz with a resolution of 1 Hz. The frequency response is relatively flat within this frequency range. The acoustic fields inside the resonators were measured by a 1/4-inch microphone (Brüel & Kjær, Type 4935) and were then transferred to the lock-in amplifier via a conditioning amplifier (Brüel & Kjær, 64 NEXUS Type 2693A).

Supplementary Note 5. Effect of loss in coupling waveguides

The coupling waveguides that connect the resonators are relatively narrow and will generate a certain amount of loss. To check the lossy effect in the coupling channels, we performed full-wave simulations on structures with different amount of loss in the coupling channels. As can be seen in Supplementary Figure 5, the bandstructures almost remain the same as we increase the loss in the coupling channels from γ_1 (a) to $2\gamma_1$ (b) and $3\gamma_1$ (c). Here γ_1 is the background loss in the resonators. Thus, the loss in the coupling waveguides has negligible effect on the real part of the dispersion and the size of the bandgap.

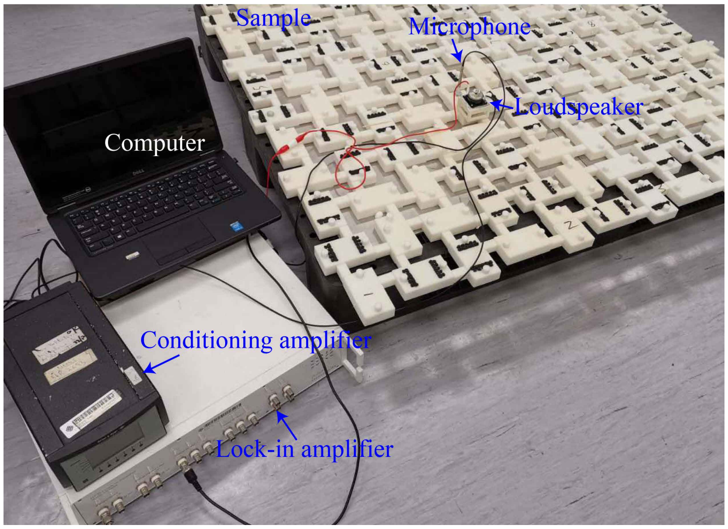

Supplementary Figure 4 Experimental setup.

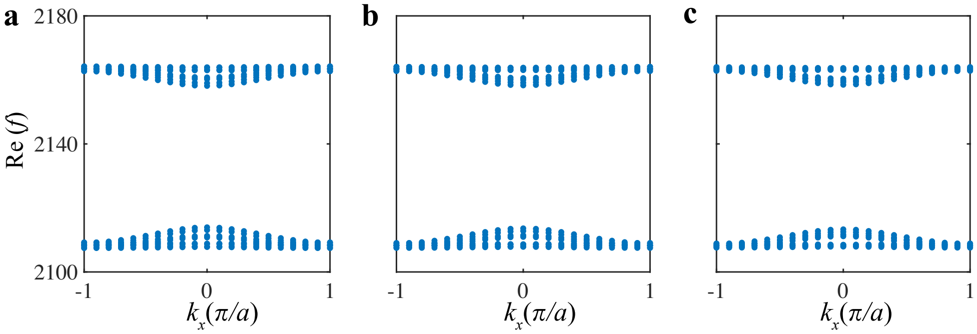

Supplementary Figure 5 Bandstructures for lattices with different losses in coupling waveguides. a-c, Bulk dispersion for acoustic lattices with loss in the coupling waveguides being γ_1 (**a**) to $2\gamma_1$ (**b**) and $3\gamma_1$ (**c**), where γ_1 is the background loss.

Supplementary Note 6. Simulations in the nontrivial lattice

Here we provide numerical simulations on the topological lattice for comparison with the experimental results shown in Fig. 3 in the main text. Firstly, the spectra in the one of the corner resonators, edge resonators and bulk resonators are numerically calculated, as denoted by the red, yellow and blue curves in Supplementary Figure 6a, respectively. Two peaks appear in the bulk spectrum, which correspond to the two branches of bulk states. Similarly, two peaks in the edge spectrum correspond to the gapped edge states. In contrast, only one peak can be observed in the corner spectrum. The spatial intensity profiles at the typical frequencies, denoted by the red (2137 Hz), yellow (2157 Hz) and blue (2165 Hz) dashed lines in Supplementary Figure 6a, are also simulated and given in Supplementary Figure 6b-d, which confirm the existence of corner, edge and bulk states. All these simulations agree with the experimental results presented in Fig. 3 in the main text.

Supplementary Note 7. Robustness of the corner states

Here we perform numerical calculations based on tight-binding model as well as real structures to study the robustness of the corner states. We consider on-site resonant frequency perturbations which should be the main source of disorder in the experiment due to fabrication error and temperature fluctuation. Here two cases are studied: disorder on all sites except the four corners (case 1) and disorder only on the four corners (case 2). In the tight-binding calculations, the disorder on site i is introduced by changing the on-site terms as $f_0(1 + df_i)$, where f_0 is the resonant frequency without disorder and df_i are random numbers uniformly distributed from $-\delta$ to δ with δ being the disorder strength. For case 1, we found that the corner states remain stable as long as the bandgap remains open (Supplementary Figure 7a). For case 2, the frequencies of the corner states shift and may coincide with bulk bands for large disorder strength (Supplementary Figure 7b). In both cases, the corner states survive for weak disorders. We also confirm above results through full-wave simulations. In the simulation, the disorder is introduced by placing a hard cylinder with random size inside the resonator (see the inset of Supplementary Figure 7c). The simulated eigenfrequencies for case 1 and case 2 are shown in Supplementary Figure 7c and Supplementary

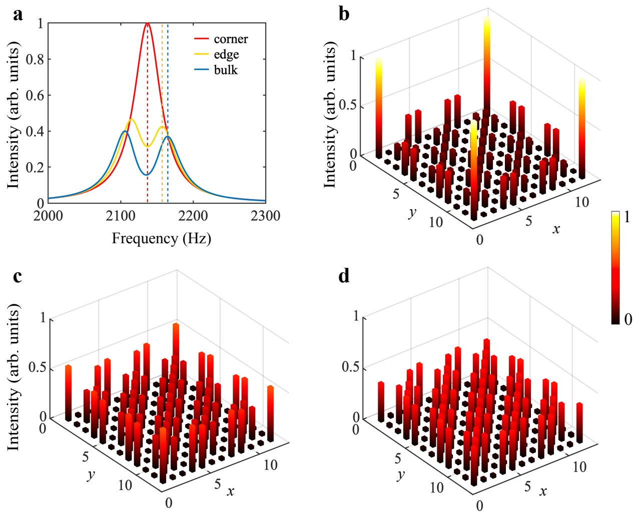

Supplementary Figure 6 Simulations in the nontrivial lattice. **a**, Simulated acoustic intensity spectra for the nontrivial sample. Red, yellow and blue curves represent the corner, edge, bulk spectra calculated at one of the resonators in the corner, edge and bulk. **b-d**, Simulated intensity profiles at the peaks of the corner, edge, bulk spectra, denoted by the red (2137 Hz), yellow (2157 Hz) and blue (2165 Hz) dashed lines in **a**, respectively.

Figure 7d, respectively. As can be seen, the corner states persist to exist and the eigenfrequencies are no longer degenerate for case 2, which are consistent with tight-binding calculations.

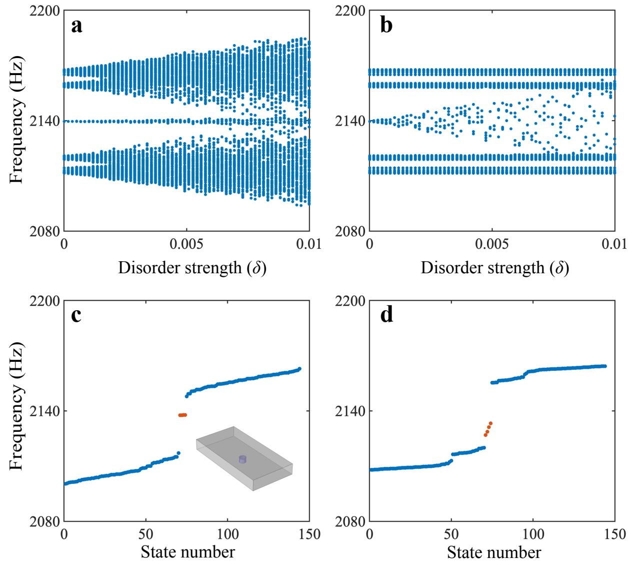

Supplementary Figure 7 Robustness of the corner states. **a-b**, Numerically calculated eigenfrequencies using tight-binding model for the finite nontrivial lattices with different onsite disorder strengths. In **a**, the disorder is introduced to all sites except the four corners. In **b**, the disorder is only introduced to the four corners. **c-d**, Simulated eigenfrequencies for a finite nontrivial acoustic lattice with onsite disorder on all sites except the four corners (**c**) and only on the four corners (**d**).

References

[1] Xue, H. *et al.* Observation of an acoustic octupole topological insulator. *Nat. Commun.* **11**, 2442 (2020).

[2] Luo, X.-W. & Zhang, C. Higher-order topological corner states induced by gain and loss. *Phys. Rev. Lett.* **123**, 073601 (2019).
